# Nanomaterial-Based Antifungal Therapies to Combat Fungal Diseases Aspergillosis, Coccidioidomycosis, Mucormycosis, and Candidiasis

**DOI:** 10.3390/pathogens10101303

**Published:** 2021-10-12

**Authors:** Angel León-Buitimea, Javier A. Garza-Cervantes, Diana Y. Gallegos-Alvarado, Macario Osorio-Concepción, José Ruben Morones-Ramírez

**Affiliations:** 1 Facultad de Ciencias Químicas, Universidad Autónoma de Nuevo León (UANL), San Nicolás de los Garza C.P. 66455, Mexico; angel.deb@uanl.edu.mx (A.L.-B.); javier.garzacn@uanl.edu.mx (J.A.G.-C.); yamilet.gallegosalvrd@uanl.edu.mx (D.Y.G.-A.); m.osoriocn@gmail.com (M.O.-C.); 2 Centro de Investigación en Biotecnología y Nanotecnología, Facultad de Ciencias Químicas, Parque de Investigación e Innovación Tecnológica, Universidad Autónoma de Nuevo León, Apodaca C.P. 66628, Mexico

**Keywords:** nanomaterials, antifungal therapies, aspergillosis, coccidioidomycosis, mucormycosis, candidiasis

## Abstract

Over the last years, invasive infections caused by filamentous fungi have constituted a serious threat to public health worldwide. *Aspergillus*, *Coccidioides*, *Mucorales* (the most common filamentous fungi), and *Candida auris* (non-filamentous fungus) can cause infections in humans. They are able to cause critical life-threatening illnesses in immunosuppressed individuals, patients with HIV/AIDS, uncontrolled diabetes, hematological diseases, transplantation, and chemotherapy. In this review, we describe the available nanoformulations (both metallic and polymers-based nanoparticles) developed to increase efficacy and reduce the number of adverse effects after the administration of conventional antifungals. To treat aspergillosis and infections caused by *Candida*, multiple strategies have been used to develop new therapeutic alternatives, such as incorporating coating materials, complexes synthesized by green chemistry, or coupled with polymers. However, the therapeutic options for coccidioidomycosis and mucormycosis are limited; most of them are in the early stages of development. Therefore, more research needs to be performed to develop new therapeutic alternatives that contribute to the progress of this field.

## 1. Introduction

Fungi constitute a diverse group of eukaryotic organisms with distinct morphological traits colonizing different ecological niches [1]. It has been estimated the presence of 3 to 5 million fungi in the environment, of which about 300 species present the ability to establish and develop infections in human beings [2,3,4]. Most of these fungal group members are considered opportunistic pathogens because they commonly infect patients with a weak immune system [4,5]. In the meantime, only a reduced number of species represent a serious threat to healthy patients [5]. The human pathogenic fungi cause various infection types, such as skin, mucosal, and invasive infections [6]. The success of pathogenic fungi to cause the disease in humans is because they tolerate high temperatures (37 °C), fast growth, invade tissues, use the host nutrients for their benefit, and evade the host immune system [3,4]. Some of the fungal genera responsible for the most frequent lethal infections are *Aspergillus*, *Cryptococcus*, *Candida*, *Coccidioides*, *Mucor*, and *Rhizopus* [5,7].

Despite the availability of traditional and modern antifungal drugs, the prevalence of mycotic infections is still increasing [8,9]. This scenario may be related to fungi’s ability to develop multi-resistance mechanisms to the existing antimycotic therapies [10]. Furthermore, the current antifungals present some drawbacks in the efficacy, efficiency, selectivity, toxicity, resistance mechanisms, and activity spectrum [11]. Contrary to other infections (bacterial or viral), those caused by fungi have been poorly explored despite the death rates each year (1.5 million) and the severe problem that is presented to the health sector [12,13]. In this context, the generation of drug delivery systems based on nanomaterials or nanoparticles (NPs) represents a potential alternative to developing newer pharmaceutical formulations to successfully combat fungal infections and overcome the fungal multi-resistance to existent drugs [14]. Nanoparticles can be synthesized from lipids, polymers, and metals [15,16,17,18]. Different research groups have determined that nanoparticles present fewer side effects, greater specificity to the infection site, do not generate drug resistance, improve antimycotic stability and solubility, and increase efficiency [15,19,20,21,22,23]. Due to these characteristics, the different nanoparticle types are considered promising biopharmaceutical systems due to their improved antifungal properties, unlike traditional drugs for treating various fungal pathogens [21]. Thus, this review will be divided into three main sections: an overview of the fungal disease, current treatment, and nanotechnology as the main tool for improving antifungal therapy.

## 2. Overview of the Fungal Disease

Over the last years, the number of invasive filamentous fungal infections has increased considerably. Immunosuppressed individuals with HIV/AIDS, uncontrolled diabetes, hematological diseases, transplantation, and chemotherapy, are more frequently susceptible to fungal infections. *Aspergillus*, *Coccidioides*, *Mucorales* (the most common filamentous fungi), and *Candida auris* (non-filamentous fungus) can cause infections in humans. They are well characterized and constitute a serious threat to public health worldwide [24]. Nanomaterials (both metallic and polymeric nanoparticles) and their applications as novel strategies for treating the most prevalent invasive filamentous fungal infections are described in this review article. In Table 1, we summarized the geographical distribution and epidemiological data of the most common filamentous and non-filamentous fungi that cause infections in humans.

### 2.1. Aspergillosis

Some molds can produce mycotoxins. Both humans and food can be contaminated with mycotoxins. Mycotoxins have various acute and chronic effects on humans, including allergies, hepatotoxicity, and cancer. *Aspergillus* is a group in the fungi kingdom belonging to the Ascomycota phylum, Eurotiomycetes class, Eurotiales order, Trichocomaceae family, and *Aspergillus* genus, and species: *niger*, *fumigatus*, *flavus*, *ochraceus* [32]. *Aspergillus flavus* (*A. flavus*) and *Aspergillus fumigatus* (*A. fumigatus*) are two ubiquitous molds capable of producing and releasing mycotoxins. Mold growth is prevalent in hospital environments, causing patient complications and economic losses [33]. *A. fumigatus* is a saprotrophic fungus, which spreads by asexual sporulation, with vegetative mycelial life in organic material in decay [34]. It can cause lung infections due to the inhalation of airborne conidia present in indoor and outdoor environments. Severe asthma with fungal sensitization, chronic pulmonary aspergillosis, and invasive pulmonary aspergillosis are also related [35,36,37]. *Aspergillus* species are the most common cause of corneal disease. Similarly, fungal keratitis, the third clinical presentation of fungal infections, is associated with saprophyte filamentous fungi, such as *Aspergillus* spp. [38]. Because of this, *Aspergillus* species are important life-threatening causes of illness, most notably in immunocompromised hosts [39].

### 2.2. Coccidioidomycosis

Coccidioidomycosis, also known as valley fever, is a systemic fungal infection caused by *Coccidioides immitis* or *Coccidioides posadasii*. *Coccidioides* genus (*immitis* and *posadasii* species) are fungi within Ascomycete phylum, Eurotiomycetes class, Onygenales order, and Onygenaceae family [40]. The two main species reside in the soil of certain parts of the Western Hemisphere [41]. Coccidioidomycosis is primarily transmitted via respiratory inhalation of airborne arthroconidia, the inhalation of a single spore may be enough to cause illness, but higher spore burdens are more likely to result in significant disease and may precipitate acute respiratory distress syndrome (ARDS) [42].

In addition to residency or travel in endemic areas, immunocompromised people by advancing age, pregnancy, cancer (especially chronic lymphocytic leukemia and non-Hodgkin lymphoma), corticosteroid therapy, diabetes, organ transplantation, or human immunodeficiency virus (HIV) infection are at significantly increased risks of contracting coccidioidomycosis [43]. Although most infected individuals do not develop any symptoms, the rest have symptoms ranging from fever, fatigue, cough, dyspnea, chest pain, and pneumonia with severe and life-threatening complications [44]. Additionally, if the infection becomes extrapulmonary, medical intervention may be necessary. Infections can disseminate to the spleen, liver, brain, bone, and many other tissues in the body [45].

### 2.3. Mucormycosis

Mucormycosis is an emerging life-threatening infection caused by various fungal species of the order Mucorales [46]. *Rhizopus* and *Mucor* represent the most common agents inducing this lethal infection [29]. *Rhizopus* is a group in the fungi kingdom that belongs to the Mucoromycota phylum, Mucoromycetes class, Mucorales order, Rhizopodaceae family, *Rhizopus* genus, and species: *microsporus* and *delemar*. On the other hand, *Mucor* is a genus of mold. It is a member of the kingdom fungi, Mucoromycetes class, Mucorales order, and Mucoraceae family [32]. The Mucorales exhibit fast growth and produce a high number of asexual spores. Humans are frequently exposed to Mucorales spores via inhalation, ingestion, or direct inoculation into a disrupted skin or an open wound [47]. Among the common clinical forms are rhinoorbital/cerebral, pulmonary, renal, gastrointestinal, cutaneous, and disseminated disease [47]. The common risk factors enabling the development of mucormycosis include uncontrolled diabetes mellitus, cancer, solid organ or bone marrow transplantation, hematological malignancy, corticosteroid treatment, and trauma and burns [48].

In recent years, an evolution in mucormycosis epidemiology has been observed with the emergence of new risk factors worldwide, including coronavirus disease (COVID-19) [49]. The COVID-19 disease pattern may differ with the severity of the disease; it can range from mild to life-threatening pneumonia with associated bacterial and fungal coinfections. Thus, people with related comorbidities and immunocompromised conditions are more likely to develop severe opportunistic infections [50]. Increasing cases of mucormycosis in patients with COVID-19 have recently been reported worldwide and in India [50,51,52].

### 2.4. Candidiasis (Candida auris)

*Candida* spp. is a diploid fungus considered an opportunistic pathogen capable of causing serious infections and compromising human health, especially in individuals with weak immune systems [31,53]. Candidiasis represents the fourth nosocomial infection with a 35 to 100% mortality rate in immunosuppressed patients [53]. Among the main types of disease generated by *Candida* include cutaneous, oropharyngeal, candidemia, vaginitis, and systemic infections [31,53]. Recent data reveals that other species and *Candida albicans* (*C. albicans*) can also cause infections. *Candida auris* (*C. auris*) is an emerging pathogenic species, distributed in almost all continents, which has attracted considerable attention due to its pathogenicity, high resistance to traditional antifungals, and rapid transmission [54]. *C*. *auris* belongs to the phylum Ascomycota and is part of the Saccharomycetes class. It is included in the Clavispora/Candida clade that belongs to the Saccharomycetales order, Metschnikowiaceae family, and Clavispora genus [55]. Recent reports indicate that most *C. auris* isolates are resistant to fluconazole, amphotericin B, and echinocandins; probably, this phenomenon is the result of the uncontrolled and prolonged use of existing use antifungals [56,57]. Furthermore, the transition from blastoconidia to filamentous growth, hydrolytic enzymes, osmotolerance, biofilm formation, cell adhesion, thermotolerance, and the change from white to opaque are some of the virulence traits that contribute substantially to the pathogenesis of *C. auris* [54,55,58].

In addition, it has been observed that this new species of *Candida* can survive for prolonged times on different environmental surfaces, as well as to disinfectants [31,55,59]. All these characteristics mentioned above allow *C. auris* to adapt and survive the various existing treatments and successfully establish an infection in a patient [31,54]. The development of new nanoparticle-based antifungals is a potential way to combat infections caused by species of the *Candida* genus.

## 3. The Current Treatment

The antifungal agents currently available for treating systemic fungal infections are amphotericin B and liposomal preparations (lipid complexes and liposomes) of amphotericin B; azoles, specially triazoles: fluconazole, itraconazole, voriconazole, posaconazole, and isavuconazole; and echinocandins [4]. Table 2 presents the recommendations for the treatment of fungal infections caused by filamentous fungi.

### 3.1. Aspergillosis

There are three classes of antifungal agents available to treat aspergillosis: polyenes, azoles, and echinocandins. The first-line treatment drug is voriconazole, followed by liposomal amphotericin B (AmB). From these, Itraconazole, voriconazole, and posaconazole are widely used for the treatment of *Aspergillus* infections. Itraconazole, posaconazole, and echinocandins are primarily used in patients with intolerance of the primary antifungal therapy. Posaconazole is also used for prophylaxis against fungal infections in patients at risk. Despite this, triazole drugs-resistance in several *Aspergillus* species is emerging continuously [60]. Mortality related to infection caused by azole-resistant fungi rises from 30–50 to 88% compared with sensitive strains [61]. Currently, the use of AmB or a combination of voriconazole or posaconazole with an echinocandin may be effective for treating *Aspergillus* infections, as azoles play a limited role in the treatment of this kind of infection [60].

### 3.2. Coccidioidomycosis

The selection of the antifungal agent depends on the infection site, the severity of the disease, and the condition of the patient, especially in those who, at the time of diagnosis, have a significantly debilitating illness [62]. Oral azole therapy is the most recommended antifungal treatment (fluconazole, itraconazole, posaconazole, voriconazole, isavuconazole). Fluconazole has become the most utilized primary therapy because it is well absorbed, has fewer drug interactions, and is less expensive than other azole options [63]. Unfortunately, adverse effects from triazoles are hepatotoxicity and cardiac toxicity due to prolongation of the QTc. Furthermore, alopecia, xerosis, and cheilitis are the most frequent side effects of fluconazole, but these follow reversible discontinuation of therapy [64]. Additionally, the overprescription of fluconazole by physicians led to an increase in resistance to azole drugs. Antifungal drug resistance appears to be due to point mutations in either drug targets or transcription factors regulating agents of the resistance [65].

Amphotericin B is used in patients with severe coccidioidomycosis infections or those who clinically fail initial therapy with oral azoles [60]. Conventional amphotericin B deoxycholate is associated with dose-limited toxicities, infusion-related reactions, and nephrotoxicity [66]. Consequently, extensive efforts have been made to reformulate amphotericin B. Considering its lipophilic properties that enable the drug’s encapsulation into liposomes or binding to lipid complexes [67].

### 3.3. Mucormycosis

The treatment of mucormycosis is challenging. It involves multiple strategies like removing the infected tissue or partially infected organs, the early administration of an optimal dose of active antifungal agent, and the utilization of different adjunctive therapies [48]. Therefore, early diagnosis is crucial to promptly initiate therapeutic interventions necessary to prevent progressive tissue invasion and its devastating sequelae, minimize the effect of disfiguring corrective surgery, and improve outcome and survival [68].

Currently, few systemic antifungals have been documented as anti-Mucorales, which mostly lack specificity and effectiveness against Mucorales. Amphotericin B (amphotericin B deoxycholate, liposomal amphotericin B, amphotericin B lipid, complex, and lipid-based amphotericin B) is the first choice for the management of mucormycosis. Lipid formulations of amphotericin B have a better therapeutic index than the conventional amphotericin B deoxycholate; however, the optimal dosage, infusion-related side effects, and high risk of nephrotoxicity are significant potentially therapy-limiting effects [46]. Posaconazole is the second effective antifungal agent followed by novel members of the azole group (triazole) like isavuconazole. Clinical studies on the efficacy of posaconazole for mucormycosis are scarce. Posaconazole could be an option as salvage therapy in patients unresponsive or intolerant to liposomal amphotericin B [69]. Isavuconazole offers some advantages, including good tolerability, a lower side effect profile, excellent bioavailability, and reduced drug-drug interaction but updated guidelines on mucormycosis are needed to reflect the current evidence and give guidance on the use of isavuconazole for this disease [70]. Thus, new strategies to prevent and promptly treat mucormycosis are urgently needed.

### 3.4. Candidiasis (Candida auris)

The main challenge to fight *C. auris* infections is the intrinsic resistance to one or more classes of marketed antifungal drugs. Most isolates of *C. auris* are highly resistant to fluconazole, but the determination of MIC also showed that some strains are resistant to all available classes of antifungal drugs [54]. Optimal treatment against *C. Auris* is not yet defined. A suggested initial therapy is the use of echinocandins, as most isolates in the United States have been susceptible to this antibiotic class [71,72,73]. Moreover, although resistant to azoles, isavuconazole was found active against a variety of *C. auris* isolates [73].

**Table 2:** Recommendations for the treatment of fungal infections caused by filamentous fungi and *Candida auris*.

Disease	Current Treatment	References
Aspergillosis	Amphotericin B, azoles (voriconazole, posaconazole, and itraconazole), and echinocandins.	[60,61]
Coccidioidomycosis	Azoles (fluconazole, itraconazole, posaconazole, voriconazole, isavuconazole) and amphotericin B.	[63,74]
Mucormycosis	Amphotericin B, posaconazole, and isavuconazole.	[69,70,75]
Candidiasis (*Candida auris*)	Echinocandins (caspofungin, micafungin, and anidulafungin) and isavuconazole	[71,72,73]

## 4. Nanotechnology in Antifungal Therapy

Nanoparticles (NPs) comprise a wide variety of particles with size at the nanoscale level that generally ranges from 1 to 100 nm [76,77]. These particles can be of different shapes and structures, for instance, cylindrical, spherical, tubular, conical, or spiral [77,78]. Currently, NPs have gained the attention of the scientific community due to their multiple potential therapeutic and diagnostic applications, such as in drugs delivery, and as a detection system for biological and chemical agents, among others [79,80,81]. In addition, these types of materials are considered excellent therapeutic alternatives because they present less toxicity, overcome multiple biological barriers, and can be covalently conjugated with hydrophobic or hydrophilic drugs and macromolecules, improving solubility and stability [81,82,83].

Considering the shape, size, and chemical and physical properties, nanoparticles can be classified as organic or polymeric, inorganic (metallic), and carbon nanoparticles [77,84]. Organic NPs, including liposomes, micelles, dendrimers, are biodegradable, not toxic, and are sensitive to heat and light [77,85]. Generally, this class of NPs represents the first choice in the biomedical area, mainly for drug delivery, due to their high efficiency and injection on specific sites [77,81,84]. Inorganic NPs integrate all those particles synthesized from metals and metal oxides. Metal nanoparticles can be commonly derived from Cadmium (Cd), Aluminum (Al), Cobalt (Co), Copper (Cu), Gold (Au), Iron (Fe), Silver (Ag), and Zinc (Zn) [77,84]. Meanwhile, metal oxide NPs result from modifying the metal particles’ properties in the oxygen presence, increasing their reactivity and efficiency [77,84]. Among the main metal oxide nanoparticles are aluminum oxide (Al_2_O_3_), iron oxide (Fe_2_O_3_), titanium oxide (TiO_2_), and zinc oxide (ZnO). Carbon nanoparticles are particles entirely synthesized carbon and classified into fullerenes, graphene, carbon nanotubes, carbon nanofibers, black carbon, and activated carbon [77,81,84].

### 4.1. Aspergillosis

Different research groups continuously study new alternatives. A promising strategy used to fight this problem is nanotechnology. An advantage of using nanomaterials is the increased surface area to volume ratio to interact with microorganisms. From metallic nanoparticles to nanoparticles made of polymers, the use of nanomaterials could lead to a variety of antimicrobial effects due to the synthesis method. Because of this, studying the antimicrobial effect of a certain nanomaterial is of utmost importance to increase the possibilities for treating microbial infections.

#### 4.1.1. Metal Nanoparticles

As an alternative to classic drugs used to fight fungal infections, the search for new treatments has become necessary. Like those made from Ag, Au, ZnO, and TiO_2_, metal nanoparticles have great potential to be used as antimicrobial agents against various microorganisms, from bacteria to fungi [17,86,87,88,89,90,91]. These metallic nanoparticles are continuously studied to overcome microbial infections/growth. Auyeung et al. [33] studied the antimold activity of commercial NPs (metallic-, Ag, Au, and ZnO NPs) against two *Aspergillus* strains, both alone and in combination as well as in paint formulations under fluorescent light. They observed that *A. flavus* showed resistance to Ag-, Au-, and a combination of metallic: Ag-NPs, but was inhibited when using metallic-NPs (100 µg/mL), ZnO NPs (20 µg/mL), and metallic: ZnO NPs (50:10 µg/mL). *A. fumigatus* showed resistance to Au- and metallic: Ag-NPs, and inhibition using Ag-NPs (10 µg/mL), metallic-NPs (100 µg/mL), ZnO NPs (20 µg/mL), and metallic: ZnO NPs (50:10 µg/mL). When ZnO, metallic- and metallic: ZnO NPs were used in paint formulations, the combination showed an antifungal activity such as amphotericin B (AmB, used as control) at half the concentration used individually, suggesting an enhanced activity when used in combination. In the study, the presence of fluorescent light generated free radicals that reacted with O_2_, H_2_O, and CO_2_ surrounding the fungi generating toxic reactive oxygen species (ROS) damaging protein, lipid, and DNA. Moreover, the authors reported that these NPs showed no toxicity in vitro against human-derived macrophages THP-1 at concentrations up to 400 µg/mL. In another study with commercial NPs, Ogar et al. [92] tested marketed AgNPs against various fungal species, including *A. fumigatus*. Treatment with 100 mg/L caused *A. fumigatus* to grow 54% compared with 100% of control. In a similar study, Xu et al. [93] assessed the antifungal activity of marketed AgNPs against various *Aspergillus* species isolated from patients with fungal keratitis. The mean MIC_90_ of these AgNPs were 1, 0.5, 0.5, and 0.5 µg/mL against *A. flavus* (61 isolates), *A. fumigatus* (11 isolates), *A. versicolor* (12 isolates), and *A. niger* (10 isolates), significantly lower than natamycin MICs of 32, 4, 32, and 8 µg/mL, respectively.

#### 4.1.2. Organic Materials-Based Nanoparticles

An essential parameter in synthesizing metallic nanoparticles for use as antimicrobial agents is the methodology used, which has a strong influence on the therapeutic properties of the NP. Because of this, the molecules/organisms used to control particle dimensions and self-organization is very important. Sayed et al. [94] synthesized two novel spinel nanoferrites (Ag_2_Fe_2_O_4_ and Ag_2_Cr_2_O_4_) by flash auto-combustion to test their antifungal activity against four *Aspergillus* species. At a 100 mg/mL concentration compared with fluconazole, Ag_2_Fe_2_O_2_ nanoferrite exhibited 1-, 0.8-, and 0.9-times lower inhibition zones against *A. flavus*, *A. fumigatus*, and *A. niger*, respectively. On the contrary, Ag_2_Cr_2_O_4_ showed 3-, 3.1-, and 2.6-times higher inhibition zones against *A. flavus*, *A. fumigatus*, *A. niger*, respectively. This behavior was also observed in the MIC determinations, where Ag_2_Fe_2_O_2_ antifungal effects were observed at 50 mg/mL, Ag_2_Cr_2_O_4_ at 12.5 mg/mL, and fluconazole at 25 mg/mL against all four fungal species. Dilshad et al. [74] observed the antimicrobial effect of citric acid- and maleic acid-capped AgNPs against *A. niger*, *A. flavus*, *A. fumigatus*, and *Fusarium solani*. The authors observed that the maleic acid-capped NPs exhibit better antifungal activity than citric acid-capped NPs. The difference could be caused by their smaller size and round shape, resulting in more surface area interacting with the fungi strains than the citric acid NPs with larger size and rough flower shape. Furthermore, NPs of smaller sizes release more silver ions (Ag^+^) than larger particles and kill more pathogens than the latter. Pandey et al. [95] synthesized AgNPs stabilized by milk proteins and tested against *A. fumigatus* and *A. ochraceus*. Milk protein synthesized AgNPs showed a higher antifungal effect against *A. ochraceus* than *A. fumigatus* at a concentration of 400 µg/disk. Selvaraj et al. [96] used a complex of 5-fluorouracil (5FU) with AuNPs (Au@5FU) for antifungal tests against *A. niger* and *A. fumigatus*. In comparison with 5FU, Au@5FU NPs showed higher inhibition zones against *A. niger* (19 mm 5FU, 29 mm Au@5FU) and *A. fumigatus* (23 mm 5FU, 32 mm Au@5FU). Similarly, Selvaraj et al. [96] used a complex of 5-fluorouracil (5FU) with AuNPs (Au@5FU) for antifungal tests against *A. niger* and *A. fumigatus*. In comparison with 5FU, Au@5FU NPs showed higher inhibition zones against *A. niger* (19 mm 5FU, 29 mm Au@5FU) and *A. fumigatus* (23 mm 5FU, 32 mm Au@5FU).

Metal nanoparticles can be added to various polymers, forming composites, increasing, or giving new properties to the polymer, such as preventing growth or adherence of harmful microorganisms [18]. In this regard, Fonseca et al. [97] studied the antimicrobial properties of poly(lactic acid) (PLA) composite with TiO_2_ NPs. They observed that light conditions have an essential role in the antifungal property of the nanocomposite. Under white light, PLA nanocomposite reduced fungal growth at 9.2 and 52.6% with 5 and 8 wt.% of NPs, respectively. This fungal reduction increased to 66.7 and 99.9 %, with 5 and 8 wt.% of NPs, respectively, when nanocomposites were exposed to ultraviolet (UVA) irradiation. Although nanocomposites presented good antimicrobial activity in the absence of irradiation, the induction of photocatalysis caused by UVA could increase the antifungal effect due to ROS generation and subsequent decomposition of the cell membrane. Leudjo Taka et al. [98] synthesized a nanosponge composite of polyurethane cyclodextrin co-polymerized phosphorylated multiwalled carbon nanotube-doped Ag-TiO_2_ nanoparticle (pMWCNT-CD/Ag-TiO_2_) and evaluated its antifungal effect against two *Aspergillus* strains. This nanocomposite had a MIC of 437.5 µg/mL against both *A. ochraceus* and *A. fumigatus*, and this value has lower than the nanosponge without dopped NPs, 1750 µg/mL. The antifungal effect is attributed to the functional groups of the fungus membrane interacting with the nanocomposite, leading to ROS production and disruption of cell wall membrane, as a probable effect of the TiO_2_ and Ag NPs dopped to the material. Mane et al. [99] synthesized Ag and Au nanocomposites using fibroin (FSNC and FGNC, respectively) obtained from *Bombyx mori*. The synthesized nanocomposites containing Ag or Au NPs showed good antifungal activity compared to fibroin material alone when applied in a disk diffusion test against *A. fumigatus*. The minimum fungicidal concentration was tested, resulting in 2 and 10 µg/mL for FSNC and FGNC, respectively. Additionally, the authors report that both FSNC and FGNC could enhance leakage of cellular components like proteins, reducing sugars, DNA and RNA. Sabira et al. [100] tested the antifungal activity of two core-shell bimetallic nanoparticles (Cu-Ag and Ag-Cu) synthesized using polyvinyl alcohol (PVA) matrix. In this study, Cu-Ag core-shell NPs exhibited a better antifungal activity against *A. fumigatus* than Ag-Cu NPs, causing an inhibition zone of 23 and 16 mm, respectively, at 0.1 M. Contrarily, Ag-Cu showed a better fungicidal effect than Cu-Ag with minimum fungicidal concentration (MFC) of 15 and 25 µg/mL, respectively, also causing damage in fungal cell wall as observed by scanning electron microscope (SEM) images. Colloidal AgNPs contained in cross-linked chitosan biguanidine (CChG/AgNPs) were tested as antifungal nanocomposite against fungi species, included *A. fumigatus* by Slama et al. [101]. Compared with the formulation without AgNPs, CChG/AgNPs showed better antifungal activity in NPs containing matter and showed almost the same inhibition zone as AmB (22.1 and 23.7 mm, respectively), both at 0.98 µg/mL. Moreover, this nanocomposite showed no toxicity in vitro when used in the MCF-7 cell line.

#### 4.1.3. Plant Extracts-Based Nanoparticles

As a green synthesis method, the use of plant extracts as reducing/capping agents is widely studied due to their low cost and environmental toxicity [23,91,102]. With a leaf extract of *Prunus cerasifera*, Jaffri et al. [103] studied the synthesis of ZnO NPs and their antifungal activity against drug-resistant *Aspergillus* strains. ZnO NPs synthesized with *P. cerasifera* leaf extract showed good growth inhibition of resistant *Aspergillus* strains. When using 10 µL, ZnO NPs caused zones of inhibition of 21.05, 23.04, 19.02, and 14.01 mm against *A. niger*, *A. flavus*, *A. fumigatus*, and *A. terreus*, respectively. These zones of inhibition were higher than the obtained when using AmB against all resistant strains. Here, the authors suggest that the antifungal effect can be attributed to the generation of surface ROS and subsequent oxidative stress. Khan et al. [104] synthesized ZnO NPs using an extract of *Trianthema prtulacastrum* as a reducing agent and tested it as an antifungal agent against *Aspergillus* strains. 100 µg/mL of ZnO NPs inhibited the growth of *A. niger*, *A. flavus,* and *A. fumigatus* by 45, 41, and 51%, respectively. These ZnO NPs caused no toxic effect against MC3T3-E1 cells, showing promising results for using the synthesized NPs to treat fungal infections. Using paper wasp (*Polister sp*) nest’s extract as a reducing agent for the synthesis of AgNPs, Lateef et al. [105] tested their antimicrobial activity against *Aspergillus* strains. AgNPs showed complete growth inhibition of *A. niger* and *A. flavus* at 100 and 150 µg/mL; meanwhile, at these concentrations, *A. fumigatus* was inhibited by 75.61%. In another study, Lateef et al. [106] synthesized AgNPs using *Petiveria alliacea* leaf extract and tested similar properties to the synthesized with a wasp nest. Here, the leaf extract synthesized AgNPs showed complete inhibition of *A. flavus* and *A. fumigatus*, but only 66.67% growth inhibition of *A. niger*. In addition, these NPs were tested for anticoagulant and thrombolytic activity. The leaf extract-synthesized AgNPs showed good anticoagulant activity. Meanwhile, using wasp nest showed suitable anticoagulant and thrombolytic activity, suggesting that these AgNPs are useful for fighting fungal infections and various biomedical applications.

#### 4.1.4. Nanoparticles Obtained from Prokaryotic/Eukaryotic Cultures

A variety of prokaryotic/eukaryotic cell cultures are widely studied for the green synthesis of NPs. El Sayed et al. [107] used *F. solani* culture as a synthesis medium of metal NPs (Ag, Cu, and ZnO). They evaluated their activity against clinical isolates of *A. awamori*, *A. fumigatus*, and *Fusarium oxysporum*. The MIC obtained for each NP was 40 and 43.3 µg/mL for AgNPs, 31.67 and 41.60 µg/mL for CuNPs, and 26.7 and 28.3 µg/mL ZnO NPs against *A. awamori* and *A. fumigatus*, respectively. Similarly, using the disk-diffusion test, Ag-, Cu-, and ZnO NPs showed higher inhibition zones against *A. awamori* than *A. fumigatus*. However, ZnO NPs showed the highest inhibition zone than Ag- and CuNPs at the same concentration. This effect can be caused due to the destruction of cell membrane integrity by the abrasive surface of Zn ONPs and ROS production [108]. Using *Penicillium expansum* culture as a synthesis method for the obtention of Se NPs, Hashem et al. [109] studied the effect of these NPs against some *Aspergillus* strains. The authors report the MIC of Se NPs as 250 µg/mL with an inhibition zone of 10.4 and 11 mm for *A. fumigatus* and *A. niger*, respectively, possibly caused by Se NPs interaction with the cell membrane, leading to physical damage and leakage of cellular compounds. Similarly, Khan et al. [110] synthesized AgNPs from *Bacillus sp.* MB353 culture and tested against *A. niger*, *A. fumigatus,* and *F. soleni*. These AgNPs showed good antifungal activity against *A. niger,* causing 94% growth inhibition at 50 µg/mL, but only 60% growth inhibition against *A. fumigatus* and 74% against *F. soleni* at the same concentration. Unfortunately, these AgNPs showed high cytotoxicity at 25 µg/mL, with cell death rates of 88% against human fibroblast cells, limiting the use of these NPs to treat fungal infections. Using a cell-free extract of *Bacillus safensis* LAU 13, Ojo et al. [111] synthesized Ag and Ag-Au NPs and evaluated their antifungal activity. The monometallic NPs caused 66.67 and 75.32% of growth inhibition against *A. niger* and *A. fumigatus*, respectively, at 200 µg/mL. Ag-Au NPs caused 83.33 and 90.78% inhibition against *A. niger* and *A. fumigatus*, suggesting that bimetallic nanoparticles could be more potent than monometallic NPs. This antifungal effect could be attributed to damage to the cell wall. Shakibaie et al. [112] synthesized Se NPs in a *Bacillus sp*. MSh-1 culture for its use as an antifungal agent against *A. fumigatus*. These Se NPs exhibited a MIC value of 100 µg/mL against *A. fumigatus* using the broth dilution method. In another study using a *Streptomyces sp*. VITSTK7 filtrate, Thenmozhi et al. [113] synthesized AgO/Ag NPs to use them as antifungal agents against *Aspergillus* pathogens. AgO/Ag NPs at a 50 µg/mL concentration showed significant antifungal, causing inhibition of 75.25, 67.22, and 62.30% against *A. fumigatus*, *A. niger*, and *A. flavus* respectively. The antifungal activity of these AgO/Ag NPs may be caused by a reduction in spore’s production as *A. niger* spores were not produced after prolonged incubation with the NPs.

#### 4.1.5. Nanoparticles-Based Drug Delivery or Controlled Drug Release Systems

Nano-formulation of conventional antifungal compounds is advantageous to control the release of the active ingredient by manipulating the outer shell of the nano-capsules, leading to better treatment of various diseases. Roy et al. [114] synthesized nanocomposites of polyethylene glycol (PEG) containing hexaconazole (N-Hexa). The resulted nanocomposite was tested against two *A. niger* strains and one *A. fumigatus* strain. After 48 h, N-Hexa caused a reduction of fungal growth from 26 to 9 and 4 mm for *A. niger* species, at 5 and 10 ppm, respectively, and a reduction from 20 to 3 and 1 mm for *A. fumigatus*. Authors reported that incorporating hexaconazole to PEG increased its water solubility, leading to a better antifungal effect than a non-encapsulated formulation. Furthermore, due to the poor bioavailability of currently approved drugs, the use of different drug delivery technology, such as hydrogels, micro-particles, NPs, and liposomes, has been proposed to enhance bioavailability and prolonged drug release. Bhatta et al. [115] studied mucoadhesive lecithin/chitosan (L/C) NPs as new drug delivery for natamycin, for antifungal activity against *C. albicans* and *A. fumigatus* compared with natamycin alone and a commercial ophthalmic suspension. The authors reported similar MIC of NPs compared with commercial suspension and natamycin when used 10, 25, and 50 µg of the active compound. The NPs, although they presented lower inhibition than natamycin, showed higher zones of inhibition when compared with the commercial formulation. In addition, the authors reported no ocular damage or clinically abnormal signs in the cornea when NPs were tested using an in vivo rabbit model. In another study, Chhonker et al. [116] proposed using L/C NPs as a drug delivery system for amphotericin B and its antifungal activity against *A. fumigatus* clinical isolate. L/C NPs showed a MIC of 0.12 µg/mL against *A. fumigatus*; meanwhile, AmB MIC was 0.06 µg/mL and Fungizone^®^, a commercial formulation, 0.25 µg/mL. Authors suggest that the difference between AmB and NPs is because of the restricted drug release caused by the L/C matrix. AmB caused a higher zone of inhibition, but NPs and commercial formulation showed no significant difference. These studies suggest that L/C NPs, used as drug delivery systems, are comparable to marketed ophthalmic suspension by showing no differences in antifungal effects. Malhotra et al. [117] encapsulated fluconazole analogs in O-alkylated dextran nanoparticles as a drug delivery system to characterize its antifungal effect against *A. fumigatus*. The analog encapsulated in O-decyl-derivatized NPs inhibited the growth of *A. fumigatus* at an effective concentration of 3.16 µg/mL. Moreover, when the analog was encapsulated in O-hexadecyl NPs, the growth inhibition was achieved at an effective concentration of 1.63 µg/mL. These inhibitory concentrations were lower than both fluconazole and its analog alone. Here, the encapsulated form has more efficient uptake by the fungus, needing lower antifungal concentration. The authors also reported minimal in vitro cytotoxicity against human erythrocytes and cell lines at antifungal concentrations. AmB was entrapped in lipid NPs (LNPs) as a nanoscale drug carrier in a study made by Jung et al. [75] to compare its antifungal effect against fluconazole-resistant *A. fumigatus* to two commercial AmB formulations (Fungizone^®^ and AmBisome^®^) both in vitro and in vivo. Here, the AmB-entrapping LNPs showed no better in vitro antifungal activity than AmBisome^®^ but higher activity than Fungizone^®^, with MICs of 0.025, 0.25, and 1 µg/mL for LNPs, AmBisome^®^, and Fungizone^®^, respectively. Meanwhile, in an in vivo immunosuppressed mice infection model using *A. fumigatus*, AmB-entrapped LNPs showed higher survival rates than AmBisome. These results are consistent with other reports where liposomal and free AmB has comparable antifungal activities against various fungi [118]. Van de Ven et al. [119] studied the antifungal in vitro and in vivo effect of AmB-loaded poly(D, L-lactide-co-glycolide (PLGA) NPs against various fungal pathogens, including *A. fumigatus*. In vitro, AmB-loaded PLGA NPs showed significantly higher antifungal activity against *A. fumigatus* than AmB, Fungizone^®,^ and AmBisome^®^ (IC_50_ of <0.03, 0.93, 0.39, and 0.63 µg/mL, respectively), as well as lower cytotoxicity against MRC-5 cells (CC_50_ of >64, 26.4, 54.3, and >64 µg/mL, respectively) and lower hemolytic activity against red blood cells (HC_50_ of >64, 4.9, 18.3, and >64 µg/mL, respectively). Similarly, the AmB-loaded PLGA NPs caused a significant reduction of *A. fumigatus* compared with Fungizone^®^ and a 2-times more efficient reduction than AmBisome when used in vivo at a dosage of 5 mg/kg. Shirkhani et al. [120] used nebulized-AmB/polymethacrylic acid (AmB-PMA) nanoparticles as a prophylaxis treatment to prevent invasive aspergillosis. In vitro assay with *A. fumigatus* showed that AmB-PMA NPs caused total inhibition of the fungi at 300 µg of AmB in PMA. Similarly, using AmB-PMA NPs at a dose of 300 µg of AmB in PMA in vivo, total inhibition of fungal growth from the lungs of infected BLAB/c mice at day +2 of infection was observed. Khames et al. [121] prepared natamycin solid lipid nanoparticles (SLNs). These SLNs were tested against *A. fumigatus*, causing an increase in the inhibition zone by 8 mm compared to natamycin alone (18 to 26 mm). The MIC measured by broth dilution assay showed a reduction of 2.5 times when natamycin was used in SLNs. Furthermore, ex vivo histopathological studies showed no sign of irritation or morphological changes of the corneal epithelium. Lakhani et al. [122] synthesized AmB loaded PEGylated nanostructures lipid carriers (AmB-PEG-NLC) for a better ocular biodistribution after topical instillation. The antifungal activity was tested in vitro against *A. fumigatus*. AmB-PEG-NLC showed an MIC of 1.25 µg/mL against *Aspergillus*, half the concentration of AmB alone (2.5 µg/mL) and the marketed formulations Fungizone^®^ and AmBisome^®^ (0.62 and 1.25 µg/mL, respectively). This AmB-PEG-NLC showed no cytotoxicity to ARPE-19 cells at a concentration range of 0.95–30 µg/mL, suggesting a good therapeutic index. Moreover, the authors report similar AmB concentration in ocular tissues when using AmB-PEG-NLC or AmBisone^®^ in vivo, making this formulation an alternative for the topical delivery of AmB.

As mentioned, studying the effects of a variety of nanomaterials increases the possibilities for treating aspergillosis. The use of green methodologies increases the bioavailability of metallic nanoparticles. These nanoparticles could be used as delivery systems of various antimicrobial compounds. In addition, the use of nanomaterials made of polymers as delivery systems is comparable to marketed formulations. The studies included here show that nanomaterials could be considered an alternative for treating fungal infections as aspergillosis. A summary of nanomaterials used in the treatment of *Aspergillus fumigatus* infections is presented in Table 3.

### 4.2. Coccidioidomycosis

A significant impact of nanobiotechnology for new formulations of conventional drugs is the possibility of designing functional drug delivery systems at the nanoscale. Thus, their pharmacokinetic and pharmacodynamics properties can be modified to optimize their pharmacological response [123]. The U.S. Food and Drug Administration has approved the use of AmB nanoformulations to treat systemic fungal infections. Three lipid formulations of amphotericin B have been developed to face its toxicity issues [124]. Amphotericin B lipid complex (ABLC, Abelcet^®^, The Liposome Co., Princeton, NJ, USA) consisting of a microscopic ribbon-like lipid structure; liposomal amphotericin B (L-AmB, AmBisome^®^, Gilead. Sciences, Sandimas, CA, USA) in which the drug is intercalated within the lipid bilayer of cholesterol-containing liposomes; amphotericin B colloidal dispersion (ABCD, Amphotec^®^/Amphocil^®^), in which the drug forms disk-shaped lipid structures with sodium cholesteryl sulfate, a naturally occurring cholesterol metabolite [125,126].

In a report by Furebring et al., severe and common side-effects were reported from 19 patients treated with amphotericin B lipid complex (Abelcet) at Huddinge Hospital, Huddinge, Sweden, and at Rigshospitalet, Copenhagen, Denmark. Fourteen patients from 19 discontinued Abelcet mainly due to renal toxicity, increase in bilirubin, erythema, and infusion-related symptoms, such as fever, chills, and severe vomiting. These results suggest that Scandinavian people might be more sensitive to Abelcet than those from the UK and the USA [127].

It has been shown that AmBisome is an effective treatment for coccidioidomycosis in humans [128]. Liposomal AmB has been successfully used as an alternative and safe option in treating disseminated coccidioidomycosis in a patient who was on steroid therapy [129]. In Mexico, a meningeal coccidioidomycosis clinical case was treated with AmBisome for nine months and no liver or kidney toxicity data were reported [130]. Furthermore, AmBisome has been employed in combination with azoles in a rare case of disseminated coccidioidomycosis (with osteomyelitis and compression of the lumbar vertebral body) [131].

Clemons et al. (2009) compared the efficacy of Abelcet and AmBisome in coccidioidal meningitis caused by *Coccidioides posadasii* in rabbits. The treated animals showed few clinical signs of infection, and the number of CFU in the brain and spinal cord was 100- to 10,000-fold lower than the control group. These data show that both antifungal agents were similarly highly effective [132]. The efficacy and toxicity of amphotericin B lipid complex or liposomal amphotericin B were evaluated in patients with severe coccidioidomycosis infections. A retrospective pre-post study design was conducted in patients between 2005 and 2014. Results showed that both formulations were equally effective in the treatment of this fungal infection. Moreover, AmBisome showed less renal toxicity than Abelcet, and therefore it is preferred to use in patients with baseline renal impairment [66]. Another study also evaluated the efficacy of lipid formulations of amphotericin B (AmB lipid complex, AmB colloidal dispersion, and liposomal AmB) compared to conventional amphotericin B in a murine systemic coccidioidomycosis model. The results demonstrated that conventional amphotericin B was more active than lipid formulations in reducing the fungal burdens. However, lipid formulations could be administrated at higher doses that were well tolerated, achieved sterilization of tissues, and prolonged survival of mice compared with no treated control [133]. Table 4 presents a summary of nanomaterials used in the treatment of *Coccidioidomycosis* infections.

As we described, coccidioidomycosis is a worrisome public health issue; however, there are some limitations of current treatment in terms of clinical efficacy and efficiency. Therefore, it is of utmost importance to apply nanotechnology for drug development, synthesize new and innovative carriers (nano-based drug delivery systems) beyond the production of amphotericin B liposomal formulations. Thus, the design and development of novel therapeutic alternatives is an emerging need.

### 4.3. Mucormycosis

The Mucorales have developed multi-resistance to current antifungals used to treat the infection, including amphotericin B, posaconazole, and isavuconazole [134,135,136,137]. This fact complicates the treatment of the disease. Thus, the development of effective and safe antifungal agents is an urgent need for the appropriate treatment of mucormycosis. The new antifungal therapies generated against some *Mucorales* species include nanoemulsions NB-201, silver nanoparticles (AgNPs), and zirconium oxide nanoparticles (ZrO2NPs). However, most of them are still in the in vitro experimental phase [138].

Silver formulations have less toxicity to human cells and are highly effective against human pathogens [90,138]. Because of this characteristic, the silver nanoparticles have attracted attention against human pathogens [139]. Fungal activity assays revealed the ability of silver nanoparticles encapsulated with β-cyclodextrin to contend against Mucorales, which caused a reduction in the *M. ramosissimus* growth [138]. This data proposes silver nanoparticles as a potential antifungal system to stop the course of mucormycosis. However, additional studies, including other *Mucorales* species, are needed to obtain more information about the antifungal properties of silver nanoparticles [138,139]. The zirconium oxide nanoparticles have also emerged as a new antifungal therapy. To date, there are few works on the antifungal activity of zirconium oxide nanoparticles. Still, it has been possible to demonstrate their ability to inhibit several *Mucor* and *Rhizopus* species [140]. The nanoemulsion NB-201 constitutes another antimycotic therapeutic option. NB-201 contains benzalkonium chloride (BZK) surfactant, which affects the fungal cell membrane killing of the organism [141,142]. BZK lacks toxicity for the human epidermis and is a common component of antiseptic creams and medical consumer products [143]. In vitro susceptibility tests revealed a high ability of NB-201 to reduce the growth of *Mucorales*, between them various *Mucor* and *Rhizopus* isolates (*M. circinelloides*, *R. microsporus* and *R. delemar*) [142,144]. This result suggests that nanoemulsion NB-201 can be used as a topical treatment for combating mucormycosis [144]. Table 5 presents a summary of nanomaterials used in the treatment of *Mucorales* infections.

Nowadays, the relationship between the antifungal properties of nanoparticles and mucormycosis remains poorly characterized. The synthesis of new antifungal drugs is crucial and urgent to counter the high mortality levels from mucormycosis in immunocompromised patients.

### 4.4. Candidiasis (Candida auris)

Currently, various nanoparticle-based antifungal therapies have been developed and are being evaluated against *C. auris*. These types of nanoscale particles constitute a new generation of antifungals and a promising strategy to overcome fungal infections.

Recent data show that silver nanoparticles have activity against nosocomial infections and multi-resistant agents [145,146,147]. Several research groups have demonstrated a high inhibitory activity of AgNPs on biofilm formation and growth of *C. auris* [146,148]. AgNPs-treated biofilms exhibit a cell wall alteration due to disruption of the *C. auris* cell wall [146]. Additional analyzes also showed an important activity of AgNPs on medical surfaces (silicone elastomers) and bandage fibers, decreasing the formation of biofilms [146]

In addition, AgNPs-functionalized fibers negatively impact the growth of the fungus even after several washes [146]. On the other hand, Ag-Cu-Co trimetallic nanoparticles exhibited strong fungicidal activity against *C. auris*, causing a reduction in growth, less cell viability, cell arrest, and damage to the mitochondrial membrane inducing the release of apoptotic markers [149]. In addition, these trimetallic particles do not exhibit toxicity and are currently in phase two of in vivo studies [149]. This fact indicates that trimetallic nanoparticles are excellent candidates for the development of antifungal agents [149]. Vazquez-Munoz et al. (2020) analyzed the antimicrobial properties of bismuth nanoparticles (BiNPs), which showed a powerful activity altering cell morphology and biofilm formation. However, further studies of the activity of BiNPs against *C. auris* are necessary [150].

Recent studies indicate that nanoparticles generating nitric oxide (NO) are capable of eliminating *C. auris* [151]. This type of nanoparticle was able to suppress the formation of biofilm and planktonic from *C*. *auris* isolates used in the study. This fact suggests that nitric oxide nanoparticles appear to be an excellent antifungal candidate to fight against this multidrug-resistant fungus [151]. Table 6 presents a summary of nanomaterials used in the treatment of *Candida auris* infections.

## 5. Conclusions

**Figure 1:**
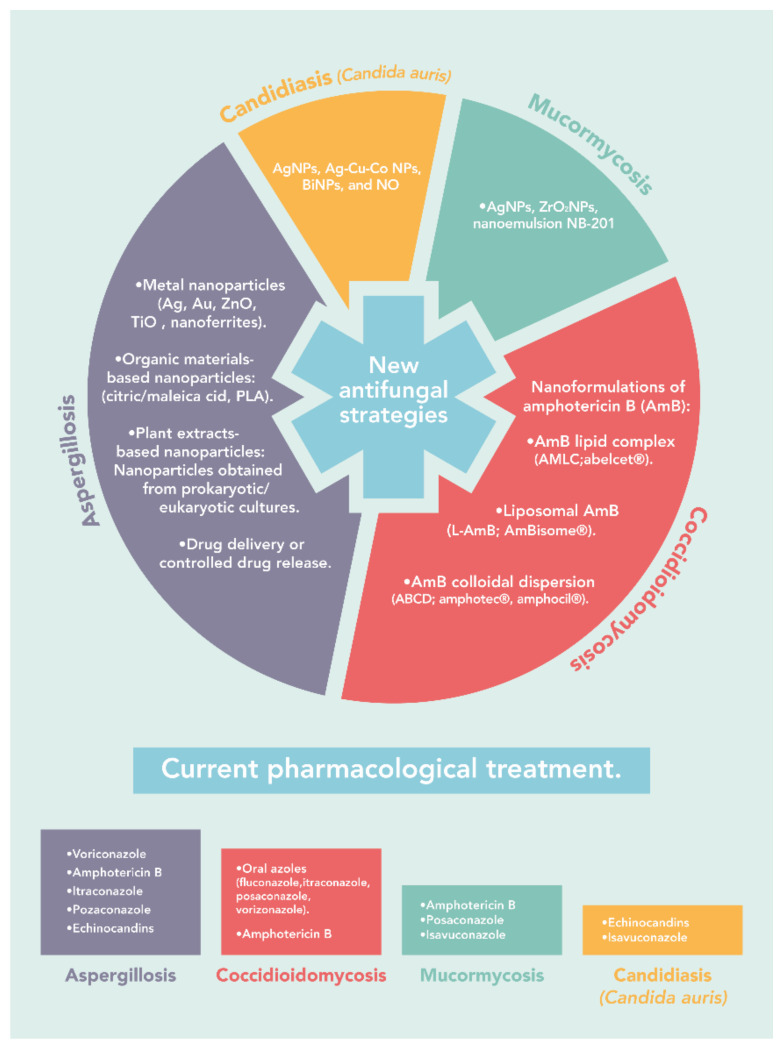
Current pharmacological treatments and the new antifungal strategies for aspergillosis, coccidioidomycosis, mucormycosis, and candidiasis caused by *Candida auris*.

The increase in invasive fungal diseases derived from the limited availability of antifungals and the development of drug resistance has shown a lack of effectiveness of current therapies. Thus, the need to find new therapeutic strategies is urgent. The continuous development of nanotechnology has favored the search and development of new and more effective therapeutic alternatives to treat invasive fungal infections. Different types of nanoformulations developed are currently being evaluated and optimized, which present improvements in the efficacy and few or null adverse that the conventional antifungals. The use of nanotechnology has allowed the development of formulations based on nanomaterials, such as metallic nanoparticles, incorporation of coating materials, complexes synthesized by green chemistry, or coupled with polymers, which can improve not only the effectiveness of the treatment but also the quality of life of the patient by reducing side effects, especially during prolonged therapies. Figure 1 summarizes the current pharmacological treatments and the new antifungal strategies for aspergillosis, coccidioidomycosis, mucormycosis, and candidiasis caused by *Candida auris*.

Various efforts are being made for the development of new drugs, as described in this review. However, the therapeutic alternatives to combat coccidioidomycosis and mucormycosis are limited or in the early development stages. The current COVID-19 pandemic has shown various factors associated with the increased prevalence of these fungal infections. Therefore, nanotechnology must continue to expand, and more research needs to be performed to develop new therapeutic alternatives that contribute to the progress of this field.

## Figures and Tables

**Table 1:** Geographical distribution and epidemiological data of the most common filamentous and non-filamentous fungi.

Filamentous Fungi	Disease	Geographical Distribution (Incidence)	Epidemiological Data	References
*Aspergillus fumigatus*	Aspergillosis	Worldwide distribution	Immunocompromised individuals with altered or weakened immune responses are able to develop aspergillosis.	[25,26]
*Coccidioides immitis and Coccidioides posadasii*	Coccidioidomycosis	Central Valley of California, desert areas of Arizona, Texas, Utah; Mexico; Central (Guatemala and Honduras), and South America (Colombia, Venezuela, Argentina, Paraguay, and Brazil).	Elderly persons, pregnant women, and members of certain ethnic groups are at risk for severe or disseminated coccidioidomycosis. Further, persons with immunodeficiency diseases, diabetes, transplant recipients, and prisoners are particularly vulnerable.	[27,28]
*Rhizopus, Mucor*	Mucormycosis	Europe (34%), Asia (31%), North/South America (28%), Africa (3%), and Australia/New Zealand (3%)	Patients with uncontrolled diabetes mellitus, cancer, solid organ or bone marrow transplantation, hematological malignancy, corticosteroids treatment, and trauma and burns are especially vulnerable to *Mucorales* infection.	[7,29]
*Candida auris* (non-filamentous fungus)	Candidiasis	Worldwide distribution	Elderly age, diabetes mellitus, recent surgery, the presence of an indwelling medical device, an immunosuppressed state, the use of hemodialysis, a neutropenic state, chronic renal disease, or the use of broad-spectrum antibiotic and/or antifungal drugs are related to *C*. *auris* infections.	[30,31]

**Table 3:** Nanomaterials used in the treatment of *Aspergillus fumigatus* infections.

Nanomaterial	Antifungal Effect	Reference
AgNPs	Growth inhibition at 10 µg/mL	[33]
	54% growth inhibition at 100 mg/L	[92]
	75.61% growth inhibition at 150 µg/mL	[105]
	Growth inhibition at 150 µg/mL	[106]
	Growth inhibition at 40 µg/mL	[107]
	60% growth inhibition at 50 µg/mL	[110]
Marketed AgNPs	90% growth inhibition at 0.5 µg/mL (clinical isolates)	[93]
AgO/Ag NPs	75.25% growth inhibition at 50 µg/mL	[113]
Ag-AuNPs	90.78% growth inhibition at 200 µg/mL	[111]
Ag_2_Cr_2_O_4_	3.1 times higher inhibition than fluconazole	[94]
Maleic acid capped AgNPs	Growth inhibition	[74]
Milk protein synthesized AgNPs	Growth inhibition	[95]
Fibroin-AgNPs	Fungicidal activity at 2 µg/mL	[99]
Ag-Cu core-shell NPs	Growth inhibition at 0.1 M and fungicidal activity at 15 µg/mL	[100]
CChG/AgNPs	Better growth inhibition than AmB at 0.98 µg/mL	[101]
CuNPs	Growth inhibition at 31.67 µg/mL	[107]
Cu-Ag core-shell NPs	Growth inhibition at 0.1M and fungicidal activity at 25 µg/mL	[100]
Au@5FU NPs	Higher inhibition than 5FU	[96]
Fibroin-AuNPs	Fungicidal activity at 10 µg/mL	[99]
TiO_2_-PLA NPs	99.9% growth inhibition at 8 wt% of NPs	[97]
pMWCNT-CD/Ag-TiO_2_ nanosponge	Growth inhibition at 437.5 µg/mL	[98]
ZnONPs	Growth inhibition at 20 µg/mL	[33]
	Higher inhibition zone than AmB (resistant strain)	[103]
	51% growth inhibition at 100 µg/mL	[104]
	Growth inhibition at 26.7 µg/mL	[107]
SeNPs	Growth inhibition at 250 µg/mL	[109]
	Growth inhibition at 100 µg/mL	[112]
N-Hexa	Growth reduction at 10 ppm	[114]
Natamicyin encapsulated L/C NPs	Similar growth inhibition than natamycin	[115]
AmB encapsulated L/C NPs	Growth inhibition at 0.12 µg/mL	[116]
AmB entrapped lipid NPs	Growth inhibition at 0.025 µg/mL	[75]
AmB loaded PLGA NPs	50% growth inhibition at 0.03 µg/mL	[119]
AmB-PMA NPs	Growth inhibition with 300 µg of AmB	[120]
AmB leaded PEG NLC	Growth inhibition at 1.25 µg/mL	[122]
Fluconazole encapsulated O-alkylated dextran	Growth inhibition at 3.16 µg/mL	[117]
Natamycin SLNPs	Better inhibition zones than natamycin	[121]

Abbreviations: CChG: Cross-linked chitosan biguanidine; 5FU: 5-fluorouracil; PLA: poly (lactic acid); pMWCNT-CD: polyurethane cyclodextrin co-polymerized phosphorylated multiwalled carbon nanotube; N-Hexa: nanocomposite-containing hexaconazole: L/C: lecithin/chitosan; AmB: amphotericin B; PLGA: poly(D, L-lactide-co-glycolide acid; PMA: polymethacrylic acid; PEG-NLC: polyethylene glycol nanostructures lipid carrier; SLNP: solid lipid nanoparticles.

**Table 4:** Nanomaterials used in the treatment of *Coccidioidomycosis* infections.

Nanomaterial	Antifungal Effect	Reference
Amphotericin B lipid complex (ABLC, Abelcet^®^)	Highly effective treatment.	[132,133]
Liposomal amphotericin B (L-AmB, AmBisome^®^)	Successfully used as an alternative and safe option of treatment.	[128,129,130,131,133]
Amphotericin B colloidal dispersion (ABCD, Amphotec^®^/Amphocil^®^)	Well tolerated and effective treatment	[133]

**Table 5:** Nanomaterials used in the treatment of *Mucorales* infections.

Nanomaterial	Antifungal Effect	Reference
Nanoemulsions NB-201	Growth inhibition	[141]
Silver nanoparticles (AgNPs),	Growth inhibition	[138]
Zirconium oxide nanoparticles (ZrO2NPs)	Growth inhibition	[140]

**Table 6:** Nanomaterials used in the treatment of *Candida auris* infections.

Nanomaterial	Antifungal Effect	Reference
Silver nanoparticles (AgNPs)	Biofilm formation inhibition, planktonic growth inhibition	[146,148]
Trimetallic nanoparticles (Ag-Cu-Co NPs)	Growth reduction, lower viability, cellular arrest, mitochondria membrane damage	[149]
Bismuth nanoparticles (BiNPs)	Affect cellular morphology, biofilm formation inhibition	[150]
Nitric oxide (NO)	Biofilm formation reduction, planktonic growth inhibition	[151]

## Data Availability

Not applicable.
